# Separating Teeth

**Published:** 1897-08

**Authors:** 


ARTICLE IV.

### SEPARATING TEETH.

A DISCUSSION.

H. W. Morgan: In wedging teeth apart, it has been the almost universal practice for the last ten years to rely on rubber. The improper use of rubber should be condemned. No one familiar with its action or its use as a means for retaining space, would for a moment think of leaving rubber between the teeth longer than from six to ten hours, and it is an inhuman and barbarious practice to leave rubber remain between the teeth for weeks at a time. Six hours in 90 per cent of the cases that come to us is sufficiently long. From six says to two weeks later the teeth should be retained in place to permit all inflammatory action to subside. In getting the cavity slightly excavated or open sufficient to retain gutta-percha, the gutta percha should not extend but slightly over the margin of the cavity, and in working down and crowding the gum away, in adjusting the rubber for the purpose of making the separation, it should not be carried far beyond the point of contact of the tooth if it would crowd the gums away and injure them. The long wearing of rubber excites inflammatory action and brings about the trouble. Packing the gutta-percha slightly over the margin of the cavity is all that is necessary to hold back, and when the rubber dam is adjusted, the gum is carried back and the rest of the operation is made as usual.

Dr. B. Holly Smith, of Baltimore: It is said that a celebrated surgeon, who lived in our town, by the name of Nathan R. Smith, advocated on all occasions a bold and free incision, and on one occasion it is said he had a little carbuncle on the shoulder which required an operation. He called in his son, and the son said, "Well, father, shall it be a bold and free incision?" He replied, "No, my son, cut just as little as you can." I feel that way with reference to the heroic cutting of dentin. If any one were to operate on my teeth, I should want them to cut just as little as possible. The fact is we do not have trouble from any bottled-up microbes in the teeth, it is the fellows on the outside that gives us trouble. The proper treatment of these cases should involve the removal of all the dentin that we see slightly decayed. Dr. Morgan has condemned the use of rubber for the purpose of separating teeth. I do not think rubber should be used to separate teeth, and there is no need of using it and having the patient come back at the end of six hours to change the rubber when other things can be used, and much more satisfactorily. Cotton with silk ligatures can be kept in position. I differ with Dr. Johnson about using gutta-percha between teeth for separation and allowing it to remain for a long time because of the very thing he said, namely, to avoid pressing away of the gum and the destruction of the contour of the gum in the inter-proximate space.

Dr. McKellops: I did not hear the whole of Dr. Black's paper, and so I am not in a position to say much about it. My friend, Dr. Morgan, has condemed the use of rubber for separating teeth, and I wish to say that there need be no fear in using rubber, provided it is placed between the teeth in a proper manner. It is not necessary to wait a week or two weeks to accomplish a separation of that kind. It all depends on the manner in which you apply it to separate the teeth. I have been using rubber all my professional life for the purpose of separating teeth, and I have yet to see a case in which I regret having used it. The same holds true with regard to the application of ligatures for separating teeth. I have had no trouble with them. It is the manner in which these things are applied and the attention that is given to them.

Dr. Gordon White, of Nashville: During all my professional career I have tried to preserve as much of the natural organs that were in a healthy condition as possible. I have always thought that the Creator could beat me in making teeth, so I have only removed the diseased portion of the tooth substance, and have not cut away very much of the dentin of late, as Dr. Black has advocated. I enjoyed his paper and the remarks that have been made on it, by which I am encouraged to extend my cutting beyond what I have done heretofore.

There is no man perhaps to whom the profession is so greatly indebted as to Dr. Black, and I for one read everything he writes with the greatest interest and profit, but I have never adopted the extensive cutting that he has advocated. Some time ago I was obliged to have a slight operation performed on myself, and I had a general surgeon, a friend of mine, do it for me. He was a gentleman for whom I had done some dental work, and he said, "Now, old fellow, I will get even with you." He did not thrust his knife in more than inch or half an inch, and I thought it was about a foot deep. I am a great advocate of not quite so much cutting as has been advocated by the gentlemen who have preceded me

Dr. J. Y. Crawford, of Nashville: I want to speak in reference to the maintenance of the interdental space, or the interproximate space, as it is now termed. Nearly twenty years ago; in formulating a program for our Tennessee Dental Society, I placed on the program the query as to whether the sixth-year molar should be preserved, and one of the best men in our State, who was to open the discussion in response to that query has long gone to his home, leaving many monuments in his community to his memory. He was a good dentist, an honest workman, and very skillful; but he said he did not see why the question should be asked; that he had been extracting six-year molars for patients susceptible to the influence of carious action, and begged to be excused from making any further remark. The members of the society then urged me to make some remarks on the subject, and the first thing I said in response to that query was that the preservation of so-called six-year molars was a precedent and a necessary consideration to properly maintain the interdental space. [Applause.] I have been impressed and instructed this afternoon as I never have before in a dental society, and I must think that if the departed spirits watch over and take an interest in the affairs of men, that the immortal Webb must be looking on this assemblage to-night, for it was he who advanced the idea that in the treatment of dental caries, the cardinal feature was to maintain the tooth in its original shape. Without that you cannot hope to preserve the interdental space.

Dr. W. H. Richards, Knoxville, Tenn.: I am here to learn, and the best way I have found for acquiring information is to ask questions. In a recent conversation with Dr. Black, I ascertained that he does not use matrices. In my reading after Dr. Black, I have come to appreciate the value of the microscope, and particularly what he says in regard to the flowing of metals. I never used to think that amalgam looked like an iceberg flowing from the center gradually out of the cavity. I have been taught by him that such is the case, and it has been constantly observed by other men in the profession that the crushing power of gold on mastication brings about the same condition. I asked him if it was not scientific that a body moves in the direction of least resistance. He says that it does. Now, then, if such is the fact, how can you expect to obtain sufficient pressure against a tooth in the act of filling it, unless you have a matrix held sufficiently firm by a screw or otherwise, so as not to disturb the pressure and prevent that flow that would likely and most naturally occur under the hammer. For my benefit and for the benefit of others, and because he stands out boldly in the profession, I would ask him to explain, if he takes the position he does, if the other is more strictly scientific.

Dr. Black (closing the discussion): I am very happy in the fact that I can get along without a matrix in filling with gold even on distal surfaces. The matrix is always in the way of making good margins with gold.

As to contouring space, what is a sphere except a contour of space, or any other form that we choose to take? Now, as regards the teeth, let us try to take care of what is left of them. It has become simply a question of expediency; the cutting away of tissue that has already become injured is not a question of sentiment. It is simply a question of expediency. How can we produce the best results? Loss of tissue does not enter into it. The arc forms of the seat bring pressure on that or that (illustrating, showing a circular form and a square form) in any direction you please, which will move the quicker? That is all I have to say. It is one of the most important items in the filling of teeth. I care little if your anchorage be slight, if you have your filling well seated it will not move, provided it is sufficiently dense. Will a dense tooth resist the progress of caries and render it slower, or will it be more rapid in a tooth less dense? I do not know except from what I might reason out. We know that chalk will dissolve more rapidly than marble, and I should suppose that a tooth less dense would dissolve more rapidly than one which is very dense. But the difference in teeth is too little for this to account for much. We know from observation and experience that the hardest teeth will be dissolved very rapidly under some conditions, while the softest teeth may be totally immune from caries.—*Dental Review.*

